# A Lossless-Recovery Secret Distribution Scheme Based on QR Codes

**DOI:** 10.3390/e25040653

**Published:** 2023-04-13

**Authors:** Jeng-Shyang Pan, Tao Liu, Bin Yan, Hong-Mei Yang, Shu-Chuan Chu

**Affiliations:** 1 College of Computer Science and Engineering, Shandong University of Science and Technology, Qingdao 266590, China; jengshyangpan@gmail.com (J.-S.P.); taoliu0201@sdust.edu.cn (T.L.); yhm1998@163.com (H.-M.Y.); 2 Department of Information Management, Chaoyang University of Technology, Taichung 413310, Taiwan; 3 College of Electronic and Information Engineering, Shandong University of Science and Technology, Qingdao 266590, China; yanbinhit@hotmail.com

**Keywords:** VCS, secure transmission, QR code, ECC

## Abstract

The visual cryptography scheme (VCS) distributes a secret to several images that can enhance the secure transmission of that secret. Quick response (QR) codes are widespread. VCS can be used to improve their secure transmission. Some schemes recover QR codes with many errors. This paper uses a distribution mechanism to achieve the error-free recovery of QR codes. An error-correction codeword (ECC) is used to divide the QR code into different areas. Every area is a key, and they are distributed to *n* shares. The loss of any share will make the reconstructed QR code impossible to decode normally. Stacking all shares can recover the secret QR code losslessly. Based on some experiments, the proposed scheme is relatively safe. The proposed scheme can restore a secret QR code without errors, and it is effective and feasible.

## 1. Introduction

The rapid development of the Internet has prompted the beginning of the information age [1,2,3,4]. As a two-dimensional image, the quick response (QR) code carries a lot of information [5]. A decoder can decode its information by scanning it. People can perform operations using QR codes [6], such as visiting a website, obtaining text, using mobile payment systems, and many more. The convenience of QR codes makes them popular. The spread of information across a network requires security [7,8,9,10]. The standard for a QR code is public, and its security on the Internet is dependent on methods such as authenticated key exchange [11,12,13,14], watermarking technology [15,16,17,18], or information hiding [19,20,21].

Compared to those, the visual cryptography scheme (VCS) [22,23,24,25,26] is suitable to encrypt QR codes.

The (n,n)-VCS distributes a secret among images [27,28,29,30]. Each share is a key. All shares perform the decoding method to reconstruct the secret. The secret will be not constructed if any share is not present. The main idea of the VCS is distribution. The secret is distributed among some images. Every share contains no information about the secret image. Naor and Shamir designed two fundamental matrices for the (k,n)-VCS (k≤n) [31]. The secret is distributed to *n* images. Receivers need to stack no fewer than *k* shares to recover the secret. The recovered image requires the human visual system (HVS) for decoding. This scheme is used to encrypt a binary image. Liu et al. proposed an extended VCS [32]. A VCS for gray-level and color images was proposed by Hou using halftone technology and the subtractive model [33]. The two schemes are pixel-expansible. Based on the random grid and halftone technology, Shyu designed a VCS for color images without pixel expansion [34]. Error diffusion was used to design a VCS [35]. Luo et al. designed a VCS to encrypt a continuous tone image [36], mainly relying on halftone technology. Liu designed a novel (t,s,k,n)-VCS [37]. Yan et al. designed a robust VCS resistant to noise in shares [38]. A color VCS was proposed for a binary image by the authors in [39]. A QR code was used in a VCS for a single-pixel image [40].

As an image, a QR code can also be encrypted with VCS to enhance its security. Fang designed a VCS for a QR code using two fundamental matrices [41]. The QR code is distributed to two shares with meaningless images. Stacking the two shares can generate a new image. This image is restored to a black-and-white QR code using post-processing. Fang’s scheme is pixel-expansible. Chow et al. used XOR to achieve a scheme without pixel expansion [42]. This scheme is an (n,n)-VCS, improved by Chen to (k,n)-VCS [43]. Since it has errors, the recovered QR code sacrifices its correction ability. A color VCS for a QR code by [44] Wan et al. used a big version of a QR code as the share to achieve a VCS [45]. The reconstructed QR code with errors was decoded using the error-correction codeword (ECC). Huang et al. designed a high-security VCS for a secret QR code based on the error-correction mechanism [46].

Fang’s scheme can restore a QR code without errors. However, it is a pixel-expansible scheme. Some schemes are not pixel-expansible, and they recover a QR code with errors. To design a non-pixel-expansible scheme to restore a QR code without errors, a new VCS is proposed to share the QR code. This paper analyzes the ECC and designs a method to divide the QR code into *n* areas. These areas are distributed into *n* shares. When all shares are stacked, the secret QR code is reconstructed losslessly. If n−1 or fewer shares are stacked, the recovered QR code will have many errors. The number of wrong codewords will exceed the capacity of the ECC. The standard decoder will not decode it. It is useless if it does not reveal the secret. The method in this paper is non-pixel-expansible. All versions of the QR code can be encrypted by it, but not other binary images.

The main contributions of this paper are as follows:

1. This paper uses the mechanism of the ECC to divide the QR code into *n* areas. Every area is distributed to *n* shares to achieve a non-pixel-expansible VCS. Compared to the pixel-expansible VCS (such as [37]), the generated share is the same size as the secret image using the proposed VCS.
2. Compared with [42], this paper can recover a QR code losslessly. The capacity of the ECC is not sacrificed in the recovered QR code.
3. There are no measures to protect itself during the transmission of the QR code. The proposed VCS provides a method to ensure the security of QR codes across the network.

The rest of the paper is structured as follows: Section 2 shows the VCS, the QR code, and the decoding stacking method. The proposed VCS is introduced in Section 3. All experiments are described in Section 4. Section 5 offers some conclusions.

## 2. Preliminaries

This section introduces the VCS (Section 2.1), the QR code (Section 2.2) and the decoding stacking method (Section 2.3). This is an abbreviation, as shown in Table 1.

### 2.1. VCS

The (k,n)-VCS was presented by Naor and Shamir [31]. A secret can be distributed among images. The secret is reconstructed when no fewer than *k* shares are stacked. Secret information about the restored image is recognized using the HVS. The decoding process does not need complex calculations. The user does not need complex computer knowledge.

Let $R_{•}$ (resp. $R_{\circ }$) be a black (resp. white) pixel block in the recovered image. The $n_{•}\left(\cdot \right)$ represents the number of black pixels. The (k,n)-VCS satisfies two conditions:

SecurityCondition: Fewer than *k* shares being stacked will satisfy $n_{•}\left(R_{•}\right)=n_{•}\left(R_{\circ }\right)$ in the recovered image.

ContrastCondition: The image generated by stacking more than k−1 shares will satisfy $n_{•}\left(R_{•}\right)>n_{•}\left(R_{\circ }\right)$.

When attackers use no more than *k* shares to stack, the generated image has no color contrast. It is a useless image, which will not reveal away secrets. When no fewer than *k* shares are stacked, the generated image has color contrast. This image is useful.

Naor and Shamir’s VCS is pixel-expansible. It shares a secret image using two fundamental matrices, $M_{•}$ and $M_{\circ }$. $M_{•}$ encrypts the black pixels. The white pixels are encrypted using $M_{\circ }$. As an example, a (4,4)-VC scheme is shown below:

k and the w are the black and white pixels, respectively. In (4,4)-VC, $M_{\circ }$ and $M_{•}$ have:

(1) $$\begin{array}{c} M_{\circ }=\left[\begin{array}{ccccccccc} w & k & k & k & k & k & w & w & w\\ w & k & w & k & k & w & w & k & k\\ w & w & k & k & k & w & k & w & k\\ w & w & w & k & k & k & k & k & w \end{array}\right],\;\\ \mathrm{and}\;M_{•}=\left[\begin{array}{ccccccccc} w & k & k & w & k & k & w & k & w\\ w & k & w & k & k & k & w & w & k\\ w & k & w & k & k & w & k & k & w\\ k & w & w & k & k & k & w & k & w \end{array}\right]. \end{array}$$

**Figure 1:**
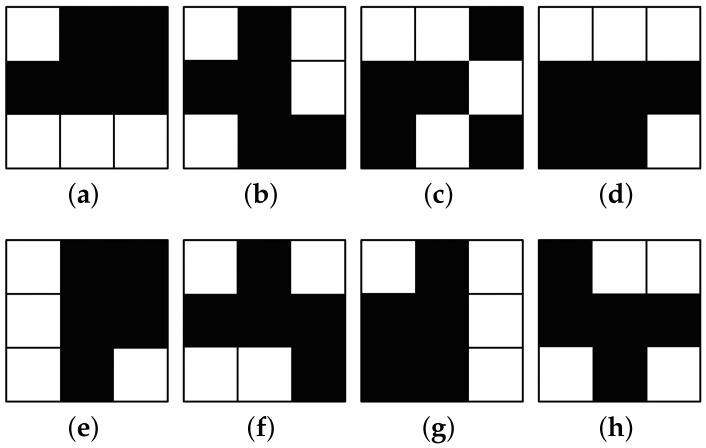
(**a**–**d**): they share the white pixels; (**e**–**h**): they share the black pixels.

Every line of the $M_{\circ }$ and $M_{•}$ represents a 3×3 pixel block, as shown in Figure 1.

When no fewer than four shares are stacked, $n_{•}\left(R_{•}\right)=n_{•}\left(R_{\circ }\right)$ is established in the recovered secret. That makes the reconstructed image non-contrasting in color. The recovered image is useless without any information. When four shares are stacked, the recovered image satisfies $n_{•}\left(R_{•}\right)=9>n_{•}\left(R_{\circ }\right)=8$. Its color has contrast, HVS can decode it, and the secret information is obtained.

For the (n,n)-VCS, only *n* shares can restore the secret. If n−1 or fewer shares are stacked, the secret will not be recovered. The decoding method of VCS is public, and the attacker knows it. If the attacker cannot obtain all shares, they cannot decode the secret. When the VCS is of the (k,n) type, the attacker needs to obtain *k* or more shares to decode the secret.

### 2.2. QR Code

QR codes are two-dimensional images carrying information [47] that are encrypted into black and white modules. D×D modules are combined into a QR code. *D* can be calculated as follows:

(2) $$\begin{array}{c} D=17+V\times 4, \end{array}$$

where *V* is the version of the QR code (V≤40). The *V* decides the number of modules. The larger *V* is, the larger *D* is.

**Figure 2:**
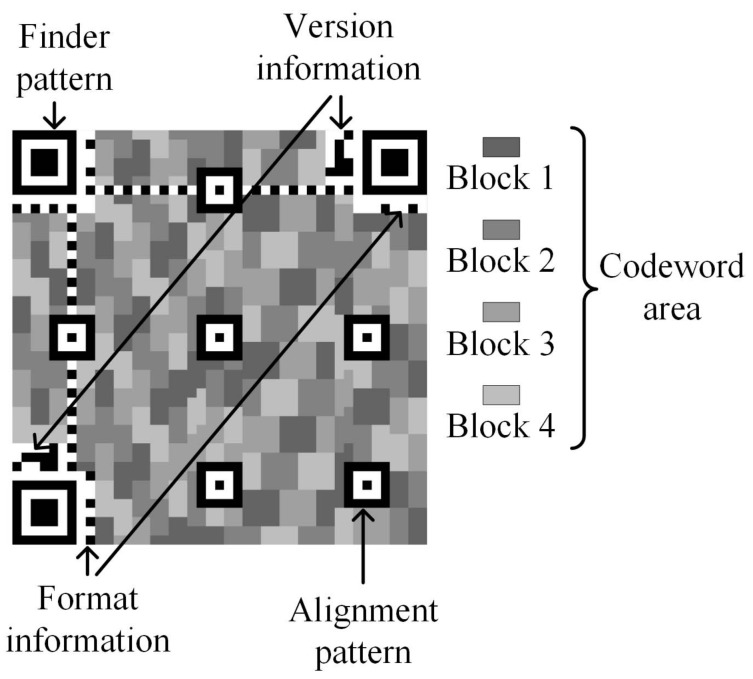
The QR code structure of version 7 and error-correction level M.

A QR code consists of a finder pattern, version and format information, alignment pattern, and codeword area, as shown in Figure 2. The codeword area is made up of blocks. Every block carries some data and error-correction codewords. Every codeword consists of 8 modules. A module is a square pattern. It is a pixel or 2×2 pixels or n×n pixels.

The finder pattern determines the location of the QR code. The ECC corrects the errors. The capacity of the ECC is over four levels, namely L, M, Q, and H. L denotes that the ECC corrects about 7% errors in the QR code. Similarly, M is 15%, Q is 25% and H is 30%. These error-correction capacities are approximations. Different QR codes can correct different numbers of codewords. The capability of each block is different. An example of the capacity of the ECC is shown in Table 2. Any module with errors will make the codeword wrong. A module can cause a codeword error. When a block has wrong codewords, the QR code cannot be decoded correctly. A few wrong modules create many wrong codewords in a block. These errors prevent the QR code from being decoded correctly.

The information is encoded as a 0–1 matrix (this process is reversible). This matrix is rendered as a black-and-white two-dimensional image. The graphical rule is that 0 (resp. 1) represents black (resp. white) modules. QR codes consist of black and white modules. When a decoder decodes QR codes, it scans them to confirm the black and white modules. All modules are then converted into a 0–1 matrix and the information is recovered. If QR codes have many errors, they cannot be decoded. For example, version 4 and error-correction level H of the QR code has nine wrong codewords in a block (it can correct eight wrong codewords; r=8 in Table 2). Therefore, it will not be decoded, and is meaningless. The QR code will lose its value. The error of the QR code refers to the black (resp. white) modules that are changed to white (resp. black) modules.

### 2.3. Stacking

This paper uses stacking to denote a specific operation performed on the colors in the image. The process for stacking the two colors is as follows:

**Definition** **1.** *Stacking: stacking (k, w) → k, stacking (w, w) → w, stacking (k, k) → k.*

When the two colors are stacked, white and white generate white (other color combinations generate black). This paper uses this operation (stacking) to achieve a secret recovery. All operations are shown in Table 3. The operation of stacking is used to recover the secret [31]. It is also the method used in this paper to restore a secret QR code.

## 3. The Proposed VCS

**Figure 3:**
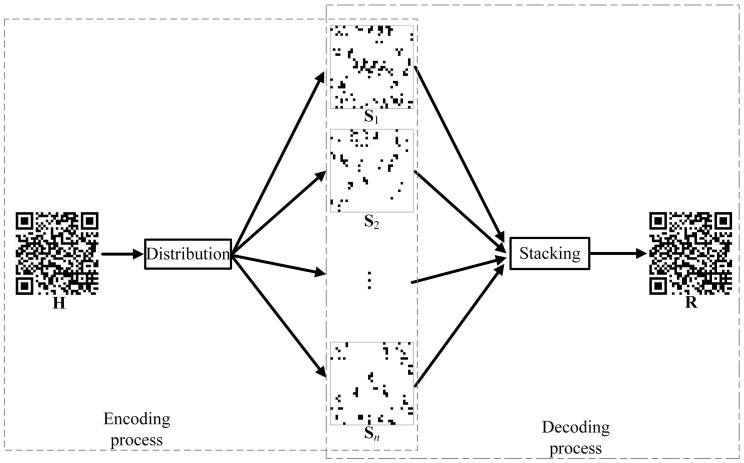
The proposed VCS.

This paper proposes an (n,n)-threshold VCS. It can distribute a secret QR code (H) to *n* meaningless images. When *n* shares are stacked, the reconstructed QR code (R) can be obtained without errors. All processes are described in Figure 3. When the receiver obtains all shares, it can stack them to recover the secret QR code. The restored QR code can then be decoded by the decoder. The secret QR code is encrypted and decrypted using a VCS. The security of its transmission across the network is improved. When all shares are obtained, an attacker can decode the QR code. If any share is missed, the attacker will not be able to decode the QR code.

### 3.1. Conditions of the Proposed VCS

Let $n_{i}^{b}\left(\cdot \right)$ show that block *b* has $n_{i}^{b}\left(\cdot \right)$ incorrect codewords. $r^{b}$ denotes the number of erroneous codewords that ECC can correct in block *b*. Every block can correct different numbers of the wrong codeword. The capacities of the different blocks are independent. Let $n_{b}\left(\cdot \right)$ be the number of blocks. The proposed VCS satisfies Condition1 and Condition2:

(3) $$\begin{array}{c} \mathrm{Stacking}\;(\overset{j}{\overset{︷}{S_{1},\cdots ,S_{i}}})\to R. \end{array}$$

(4) $$\begin{array}{c} \left\{\begin{array}{c} \mathrm{Condition}\;1:\\ \mathrm{When}\;j<n,\exists \;b\;\left(b\in \left[1,\;n_{b}\left(H\right)\right]\right)\;\mathrm{makes}\;n_{i}^{b}\left(R\right)>r^{b}.\\ \mathrm{Condition}\;2:\\ \mathrm{When}\;j=n,\forall \;b\;\left(b\in \left[1,\;n_{b}\left(H\right)\right]\right)\;\mathrm{makes}\;n_{i}^{b}\left(R\right)\leq r^{b}. \end{array}\right. \end{array}$$

When $\exists \;b\;\left(b\in \left[1,\;n_{b}\left(H\right)\right]\right),\;n_{i}^{b}\left(R\right)>r^{b}$ is satisfied in the R, the number of the incorrect codeword exceeds $r^{b}$. The R is hard to decode. Secret information will not be revealed. If the R satisfies $\forall \;b\;(b\in \left[1,\;n_{b}\left(H\right)\right])$, $n_{i}^{b}$$\left(R\right)\leq r^{b}$, it is decoded using the decoder, and the secret is obtained.

Every share is a key to restoring the secret QR code. If any share is missing, the restored QR code will have many errors. The number of wrong codewords is exceeded by the capacity of the ECC. This wrong QR code cannot be decoded using the standard decoder. The security of the VCS for the QR code is therefore ensured.

### 3.2. Encryption and Decryption Processes

To restore a QR code without errors, the white module in the QR code must also be the white module in the share. The black module of the QR code is a black module or white module in the share. Therefore, only the black modules are processed in the VCS.

Every block has error-correction codewords, which correct incorrect codewords. A QR code with more than $r^{b}$ wrong codewords will be not decoded. Therefore, any two shares need to recover more than $r^{b}$ codewords. If any share is not obtained, the recovered QR code will have more than $r^{b}$ wrong codewords. ECC can correct up to about 30%. To be more secure, when a share is missing, about half of the codeword will be wrong in a certain block of the restored QR code. Any two shares can restore modules of at least *d* codewords (the codeword is restored or the part of the codeword is restored) in every block. The *d* can be calculated by:

(5) $$\begin{array}{c} d=\left\lfloor \frac{c}{2}\right\rfloor , \end{array}$$

where a block has *c* codewords of the data and error correction. When *c* has different values in a QR code, it takes the minimum value. We use *d* and ECC to divide the QR code into area $Q_{•}^{l}$ and area ${\hat{Q}}_{•}^{l}$ (l=1,2,⋯,n).

Every area should include modules that belong to many codewords. For example, one or two modules per codeword are in each area. The module of a codeword is wrong, and this codeword is wrong. Therefore, an area is missed that will cause many codeword errors. If these wrong codewords are in a block, the QR code will not be decoded.

#### 3.2.1. The Generation of $Q_{•}^{l}$

The area of the codeword is divided into area $Q_{•}^{l}$ (l=1,2,⋯,n). The area $Q_{•}^{l}$ is generated in two cases as follows:

Case 1, When $n\in [2,\;2n_{b}\left(H\right)]$:

If $n\leq n_{b}\left(H\right)$, the $area\;Q_{•}^{l}$ is generated as follows:

𝓪. We select *n* blocks randomly. The black modules of every selected block form the area $Q_{•}^{l}$, where l=1,2,⋯,n.

𝓫. The area of all unselected black modules in each block is added to area $Q_{•}^{n}$.

For example, n=3, $n_{b}\left(H\right)=4$ and selected blocks are block 1-block 3. Area $Q_{•}^{1}$, $Q_{•}^{2}$ and $Q_{•}^{3}$ are the area of all black modules in block 1, block 2 and (block 3 and block 4), respectively.

If $n>n_{b}\left(H\right)$, the $area\;Q_{•}^{l}$ is generated as follows:

𝓪. We select $2n_{b}\left(H\right)-n$ blocks randomly. The black modules of every selected block form the area $Q_{•}^{l_{1}}$, where $l_{1}=1,\;2,\;\cdots ,\;2n_{b}\left(H\right)-n$.

For example, $2n_{b}\left(H\right)-n=3$ and selected blocks are block 1-block 3. Area $Q_{•}^{1}$-$Q_{•}^{3}$ is the area of all black modules in block 1-block 3, respectively.

𝓫. The area of all black modules (*d* different unselected codewords in every unselected block) forms the area $Q_{•}^{l_{2}}$ ($l_{2}=2n_{b}\left(H\right)-n+1,\;2n_{b}\left(H\right)-n+2,\;\cdots ,\;n$). If a block does not have *d* different codewords, it will select the next block. There is no intersection for any subset in area $Q_{•}^{l_{2}}$.

For example, $n_{b}\left(H\right)-\left(2n_{b}\left(H\right)-n\right)=1$ and an unselected block is block 4. Area $Q_{•}^{4}$ and area $Q_{•}^{5}$, respectively, consist of all black modules in *d* different codewords. They satisfy area $Q_{•}^{4}\cap \mathrm{area}\;Q_{•}^{5}=⌀$. These codewords are all from block 4.

𝓬. The area of all unselected black modules (every block) is added to area $Q_{•}^{n}$. The $\mathrm{area}\;Q_{•}^{l}=\mathrm{area}\;Q_{•}^{l_{1}}\cup \mathrm{area}\;Q_{•}^{l_{2}}$, which l=1,2,⋯,n.

Case 2, When $n>2n_{b}\left(H\right)$:

We sort the number of black modules of each codeword from small to large in block *b*. Area $A_{•}^{b}$ consists of the last *d* codewords in the sequence in block *b*. Area ${\hat{A}}_{•}^{b}$ is made up of the rest of the codewords in block *b*. The minimum number of black modules for every codeword in area $A_{•}^{b}$ (resp. ${\hat{A}}_{•}^{b}$) is denoted by $n_{1}$ (resp. $n_{2}$). The value of *b* is $b=1,\;2,\;\cdots ,\;n_{b}\left(H\right)$. The biggest value ($n_{m}$) of *n* is:

(6) $$\begin{array}{c} n_{m}=\sum_{b=1}^{n_{b}\left(H\right)}\left(n_{1}+n_{2}\right). \end{array}$$

When $n>n_{m}$, the proposed scheme is not suitable and $n\in [2,\;n_{m}]$.

Area $Q_{•}^{l}$ generates rules as follows:

Rule 1:

(7) $$\begin{array}{c} \left\{\begin{array}{cc} \mathrm{Area}\;Q_{•}^{l_{1}}=\mathrm{Area}\;{\hat{A}}_{•}^{l_{1}}, & \mathrm{if}\;l_{1}=1,\;2,\;\cdots ,n_{b}\left(H\right),\\ \mathrm{Area}\;Q_{•}^{l_{1}}=\mathrm{Area}\;A_{•}^{l_{1}-n_{b}\left(H\right)}, & \mathrm{if}\;l_{1}=n_{b}\left(H\right)+1,\;n_{b}\left(H\right)+2,\;\cdots ,\;2n_{b}\left(H\right). \end{array}\right. \end{array}$$

Rule 2: Select a black module from each codeword (these codewords are from $\mathrm{area}\;Q_{•}^{l_{1}}$, where $l_{1}$ is a random number from 1 to $2n_{b}\left(H\right)$) to perform $n-2n_{b}\left(H\right)$ times to form area $Q_{•}^{l_{3}}$, where $m_{•}^{b},\;{\hat{m}}_{•}^{b}>\;1$ and $l_{3}=2n_{b}\left(H\right)+1,\;2n_{b}\left(H\right)+2,\;\cdots ,\;n$. The selected modules are deleted in area $Q_{•}^{l_{1}}$ each time.

For example, $n-2n_{b}\left(H\right)=3$. Areas $Q_{•}^{2n_{b}\left(Q\right)+1}$, $Q_{•}^{2n_{b}\left(Q\right)+2}$ and $Q_{•}^{2n_{b}\left(Q\right)+3}$ are made up of all black modules in the first, second, and third to be chosen, respectively. The selected modules are deleted in the area $Q_{•}^{l_{1}}$.

Rule 3: $\mathrm{Area}\;Q_{•}^{l}=\mathrm{area}\;Q_{•}^{l_{1}}\cup \mathrm{area}\;Q_{•}^{l_{2}}\cup \mathrm{area}\;Q_{•}^{l_{3}}$, which l=1,2,⋯,n.

#### 3.2.2. The Generation of Area ${\hat{Q}}_{•}^{l}$

The remaining black module (except for the area of the codeword) is divided into area ${\hat{Q}}_{•}^{l}$. The process is as follows:

𝓪. Let area $H_{•}$ (resp. area $H_{\circ }$) be black (resp. white) modules of H. All black modules (except for the $\mathrm{area}\;Q_{•}^{l}$) form the $\mathrm{area}\;{\hat{Q}}_{•}$ in area $H_{•}$. $\mathrm{Area}\;H_{•}=\mathrm{area}\;{\hat{Q}}_{•}\cup \mathrm{area}\;Q_{•}^{l}$. The $n_{•}\left(\cdot \right)$ is the number of black modules.

𝓫. When $n\geq n_{•}\left({\hat{Q}}_{•}\right)$, select $n_{•}\left({\hat{Q}}_{•}\right)$ different modules of the area ${\hat{Q}}_{•}$ to form area ${\hat{Q}}_{•}^{l_{1}}$, where $l_{1}=1,\;2,\;\cdots ,\;n_{•}\left({\hat{Q}}_{•}\right)$. Select $n-n_{•}\left({\hat{Q}}_{•}\right)$ modules of the area ${\hat{Q}}_{•}$ to form area ${\hat{Q}}_{•}^{l_{2}}$, where $l_{2}=n_{•}\left({\hat{Q}}_{•}\right)+1,\;n_{•}\left({\hat{Q}}_{•}\right)+2,\;\cdots ,\;n$. The selected modules should not be repeated where possible. Area ${\hat{Q}}_{•}^{l}=\mathrm{area}\;{\hat{Q}}_{•}^{l_{1}}\cup \mathrm{area}\;{\hat{Q}}_{•}^{l_{2}}$, where l=1,2,⋯,n. Every area is made up of a black module in the area ${\hat{Q}}_{•}^{l}$.

When $n<n_{•}\left({\hat{Q}}_{•}\right)$, we select $\left\lfloor \frac{n_{•}\left({\hat{Q}}_{•}\right)}{n}\right\rfloor$ black modules (not repeating) from area ${\hat{Q}}_{•}$ for *n* times to form area ${\hat{Q}}_{•}^{l}$ which l=1,2,⋯,n. The unselected area of all the black modules from the area ${\hat{Q}}_{•}$ is added to area ${\hat{Q}}_{•}^{n}$. For example, area ${\hat{Q}}_{•}^{1}$ is the area of $\left\lfloor \frac{n_{•}\left({\hat{Q}}_{•}\right)}{n}\right\rfloor$ black modules. Area ${\hat{Q}}_{•}^{n}$ is the area of several black modules (the number is no less than $\left\lfloor \frac{n_{•}\left({\hat{Q}}_{•}\right)}{n}\right\rfloor$).

#### 3.2.3. The Encryption Process of the Proposed VCS

H is distributed to *n* shares. The encoding of the proposed VCS for H is:

Step 1: H is divided into area $H_{\circ }$, area ${\hat{Q}}_{•}^{l}$ and area $Q_{•}^{l}$ by Section 3.2.1 and Section 3.2.2.

Step 2: Generate *n* shares as follows:

(8) $$\begin{array}{c} \left\{\begin{array}{c} S_{l}\;\left(x,\;y\right)\leftarrow k,\;\mathrm{if}\;H\;\left(x,\;y\right)\in \mathrm{area}\;{\hat{Q}}_{•}^{l},\\ S_{l}\;\left(x,\;y\right)\leftarrow k,\;\mathrm{if}\;H\;\left(x,\;y\right)\in \mathrm{area}\;Q_{•}^{l},\\ S_{l}\;\left(x,\;y\right)\leftarrow w,\;\mathrm{others}. \end{array}\right. \end{array}$$

Here, $S_{l}\;\left(x,\;y\right)\leftarrow k$ (resp. $S_{l}\;\left(x,\;y\right)\leftarrow w$) represents the color of $S_{l}\;\left(x,\;y\right)$ is modified by black (resp. white).

All processes are shown in Algorithm 1. The secret QR code is divided into several areas. Every area has some black modules in a certain block. If this area is missed or is not used to restore the QR code, this restored QR code will have errors. These errors will cause the restored QR code not to be decoded by the standard decoder. All areas are distributed by *n* shares to share the secret QR code.
**Algorithm 1** The distribution algorithm**Input:**    H, the threshold *n*, area ${\hat{Q}}_{•}^{l}$, and area $Q_{•}^{l}$ (l=1,2,⋯,n). **Output:**    These *n* shares: $S_{1},\;S_{2},\;\cdots ,\;S_{n}$.
1:**for** *x* from 1toD **do**2:   **for** *y* from 1toD **do**3:     /* H consists of D×D modules.*/4:     **if** $H\;\left(x,\;y\right)\in \;\mathrm{area}\;{\hat{Q}}_{•}^{1}\;\mathrm{or}\;\mathrm{area}\;Q_{•}^{1}$ **then**5:        $S_{1}\;\left(x,\;y\right)\leftarrow k$.6:     **else**7:        $S_{1}\;\left(x,\;y\right)\leftarrow w$.8:     **end if**9:     **if** $H\;\left(x,\;y\right)\in \;\mathrm{area}\;{\hat{Q}}_{•}^{2}\;\mathrm{or}\;\mathrm{area}\;Q_{•}^{2}$ **then**10:        $S_{2}\;\left(x,\;y\right)\leftarrow k$.11:     **else**12:        $S_{2}\;\left(x,\;y\right)\leftarrow w$.13:     **end if**14:     ⋮15:     **if** $H\;\left(x,\;y\right)\in \;\mathrm{area}\;{\hat{Q}}_{•}^{n}\;\mathrm{or}\;\mathrm{area}\;Q_{•}^{n}$ **then**16:        $S_{n}\;\left(x,\;y\right)\leftarrow k$.17:     **else**18:        $S_{n}\;\left(x,\;y\right)\leftarrow w$.19:     **end if**20:   **end for**21:**end for**
**Output:**
$S_{1},\;S_{2},\;\cdots ,\;S_{n}$


#### 3.2.4. The Decryption of the Proposed VCS

The decryption operation of this paper is as follows: the receiver needs to obtain *n* shares. These *n* shares are stacked to reconstruct the QR code. Its operation is:

(9) $$\begin{array}{c} \mathrm{Stacking}\;(S_{1},\;S_{2},\;\cdots ,\;S_{n})\to \;R. \end{array}$$

R is completely recovered. Its information is obtained using the decoder. When the receiver obtains all the shares, it needs to stack all the shares to recover the secret QR code. Each share contains nothing. They are meaningless.

### 3.3. Analysis of the Proposed VCS

This paper proposes an (n,n)-VCS. Use the mechanism of the ECC to ensure that no more than *n* shares can reconstruct the secret image losslessly. The number recovering the codeword exceeds the capacity of the ECC by every share. There is about half the number of the wrong codewords in the restored image in a specific block when the number is less than *n*, using Equation (5). The ECC of the QR code cannot correct half the codeword in every block. Therefore, the proposed scheme is relatively safe. When the attacker obtains n−1 or fewer shares, they can know that the secret image is a QR code. However, they cannot decode the secret QR code. The attacker does not obtain the secret.

If the attacker obtains n−1 shares, they will stack them to generate an image that resembles a QR code. However, it will not be decoded. Some codewords have one or many wrong modules that will cause these codewords to be wrong. The errors refer to some black modules being white. If a module is wrong, it makes a codeword wrong. It is hard to confirm which white module is wrong in a recovered image. The security is mainly reflected in the following points:

1. This new image has more wrong codewords than it can correct (n−1 shares). Therefore, it cannot be decoded. The attacker will not obtain the secret.
2. The attacker knows some white modules are wrong. However, they cannot determine which areas are wrong. The errors are scattered throughout the QR code.

Due to the particularity of the QR code, this proposed scheme is secure. These n−1 shares are used to recover the wrong QR code, which cannot be decoded. All recovered black modules are right, no matter how many shares. The number of wrong modules is very small, but they are mixed with the correct ones. There are many white codewords in the QR code. The right white modules and the wrong white modules are mixed, and are difficult to correct by any means.

This paper uses the mechanism of the ECC to design a QR code. Every share can restore a certain number of codewords. This number will exceed the number that the ECC can correct. Therefore, the number of shares is connected to the number of codewords. The *n* is limited in the proposed (n,n)-VCS and $n\in [2,\;n_{m}]$.

This paper designs a scheme for a QR code. When a QR code is transmitted across a network, it can be obtained by an attacker. The standard of the QR code is public. Therefore, this QR code can easily be decoded. When using the proposed scheme to transmit the QR code, the attacker needs to obtain all the shares to restore the QR code. The proposed scheme enhances the security of the QR code on the Internet.

## 4. Experiments

The designed QR code follows ISO standard [48] and the Zxing library [49]. All experiments are introduced in this section.

### 4.1. The Simulated Experiment of the Proposed VCS

**Figure 4:**
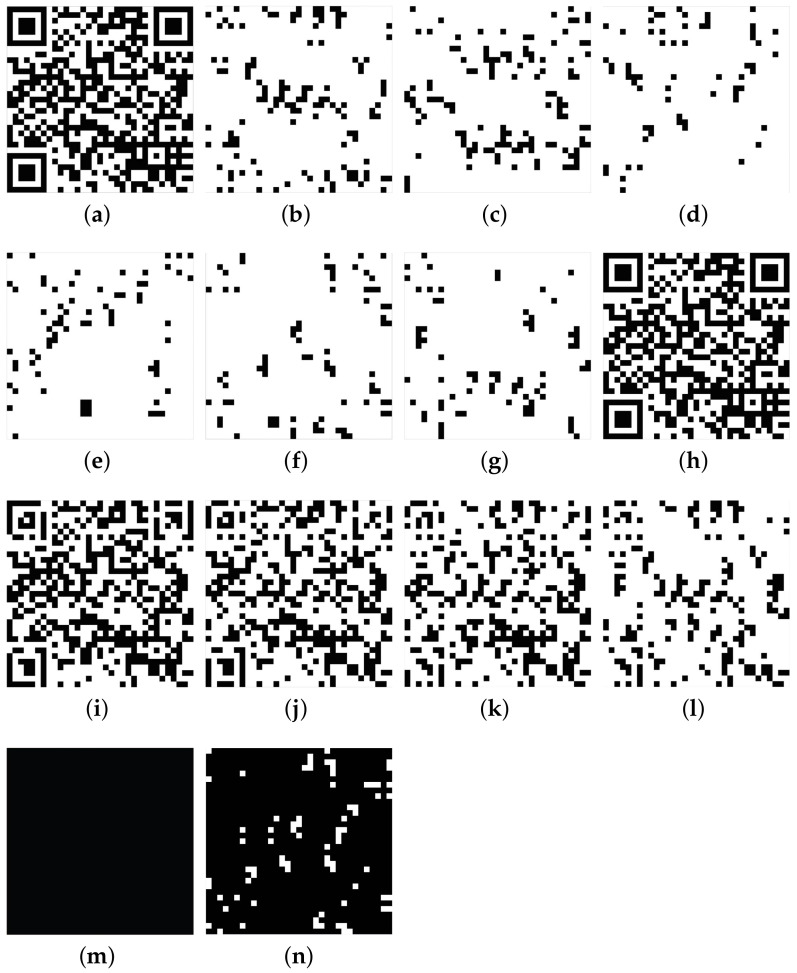
(**a**): H; (**b**): $S_{1}$; (**c**): $S_{2}$; (**d**): $S_{3}$; (**e**): $S_{4}$; (**f**): $S_{5}$; (**g**): $S_{6}$; (**h**): Stacking ($S_{1},\;S_{2},\;S_{3},\;S_{4},\;S_{5},\;S_{6}$); (**i**): Stacking ($S_{1},\;S_{2},\;S_{3},\;S_{4},\;S_{6}$); (**j**): Stacking ($S_{1},\;S_{2},\;S_{3},\;S_{6}$); (**k**): Stacking ($S_{1},\;S_{2},\;S_{6}$); (**l**): Stacking ($S_{1},\;S_{6}$); (**m**): The same between H and Figure 4h (R), where black modules denote the same; (**n**): The difference between Figure 4a,i.

Version 4 and the error-correction level H (4-H) of the QR code are adopted in this section. The experimental results are shown in Figure 4.

The H (Figure 4a) can be distributed to six shares (Figure 4b–g) when n=6. When six shares are stacked, the QR code will be recovered without errors (Figure 4h). Five or fewer shares can restore an image that resembles a QR code by HVS (Figure 4i–l). However, a standard decoder cannot decode them, and they are all useless images.

Table 4 shows some experimental results. The experiment is tested with Figure 4h–l. The decoder is a standard decoder. The results show that Figure 4 is decoded and Figure 4i–l cannot be decoded. These results verify that the proposed scheme is safe.

When stacking different numbers of shares, the reconstructed QR code has different numbers of wrong codewords, as shown in Table 5. Six shares can fully recover the QR code. If a QR code is 4-H, it can correct eight codewords in every block. If the number of stacked shares is fewer than six, the recovered QR code has some errors. The number of wrong codewords is more than eight in a certain block. The restored QR code will not be decoded by the standard decoder. When the number is less than six, the number of incorrect codewords is greater than the capacity of ECC. A standard decoder cannot decode this recovered QR code.

The proposed scheme can fully reconstruct the QR code. It is an (n,n)-VCS. We stack fewer than *n* shares to recover the image without decoding it (similar to a QR code). The error exceeds the capacity of ECC. The reconstructed QR code is a useless image. The secret will not be revealed. The restored image does not have helpful information from HVS. These experiments show that the proposed VCS is relatively safe.

Figure 4i and the secondary data in Table 5 (Stacking ($S_{1},\;\cdots ,\;S_{4},\;S_{6}$) ⋯) show that the proposed scheme is relatively safe. Figure 4i can be observed as resembling a QR code. It has more wrong codewords than can be corrected in block 2 (13 codewords are wrong in Table 5). This wrong QR code can not be decoded using the decoder. The secret will not be revealed. It is hard to determine what areas are wrong in Figure 4i except for the finder pattern. Therefore, it is hard to do brute force attacks to reveal the secret.

This paper designs an (n,n)-VCS for the QR code. When *n* shares are used to restore the secret, the secret QR code can be recovered losslessly. This can be decoded by a standard decoder. If n−1 or fewer shares are used to recover the secret, the restored QR code will have errors. A few wrong modules cause many codeword errors. The recovered QR code cannot be decoded by the standard decoder. The proposed scheme is relatively secure. This recovered QR code is deserved by HVS. However, it cannot be decoded and will not reveal anything.

Figure 4, Table 4, and Table 5 show that all shares can recover the QR code without errors. The restored QR code with n−1 shares cannot be decoded by the standard decoder. These results prove that the proposed scheme is feasible and secure.

### 4.2. Comparison of Different Schemes

The proposed VCS is non-pixel-expansible. It can completely reconstruct QR codes. Table 6 introduces the comparison result. Compared with the proposed VCS, this is a pixel-expansible scheme [41]. The two schemes belong to two non-pixel-expansible VCSs [42,43]. However, they can recover the secret image with errors. Compared with these two VCSs, the proposed VCS cannot reconstruct a QR code losslessly. Wan et al. used a QR code to share a secret QR code [45]. The secret QR code version met certain conditions. This scheme does not apply to all versions of QR codes. The proposed scheme can encrypt all versions of QR codes. This is a non-pixel-expansible scheme [46]. It can produce meaningful shares. Compared to the proposed VCS, the restored image has some errors. Compared with the pixel-expansible scheme ([41]), the proposed VCS has low time complexity.

The proposed scheme has some advantages. It can reconstruct a QR code losslessly. Moreover, its size is the same in both secret images and shares. This paper obtains a VCS for a QR code without pixel expansion. However, the proposed VCS has the shortcoming that the generated shares are meaningless images.

The scheme belongs to a (2,2)-threshold scheme [41]. It can recover a QR code without errors. It is hard to extend the (n,n)-threshold scheme. These schemes are (n,n) schemes [42,45,46]. However, they cannot restore the secret QR code with errors. To solve the above disadvantages, this paper proposes a new VCS for the QR code. The proposed scheme can recover the QR code without errors. It belongs to a (2,2) scheme. This scheme is a scheme without pixel expansion. The time complexity is O(2D×2D) in [41]. The time complexity of the proposed scheme is O(D×D). The proposed scheme is lower than Fang’s scheme in terms of time complexity.

### 4.3. Analysis and Discussion

This paper uses the mechanism of the ECC to design a VCS for a QR code. A secret QR code can be recovered without errors using all shares. The proposed scheme is an (n,n)-VCS. It will be improved to design a (k,n)-VCS in the future. Moreover, the proposed scheme generates meaningless shares. That would be difficult for a receiver and sender to manage. In the future, the authors will design a new VCS that can generate meaningful shares for a QR code.

## 5. Conclusions

Using the characteristics of the ECC, this paper proposes a method for regional partitioning. A QR code is divided into *n* areas. These areas are distributed to *n* shares in a non-pixel-expansible method. If a share is missing, many wrong codewords will exceed the amount that the ECC can correct. A recovered QR code is not decoded, and the secret will not be revealed. Stacking all the shares can completely reconstruct the QR code, and a standard decoder can decode it. The capacity of the ECC will not be sacrificed. The proposed VCS is an (n,n)-VCS, which will be modified to a (k,n)-VCS later. The share is meaningless in this paper. In the future, a new scheme will generate meaningful shares. The proposed scheme is not resistant to outside attacks. A scheme that can resist external attacks will also be designed.

## Figures and Tables

**Table 1:** Denotation of symbols.

Abbreviation	Explanation
H	The secret image
R	The recovered image
$S_{i}$	Share
•	Black
∘	White
k	Black pixel
w	White pixel
n(·)	The function of the quantity
*n*	Natural number (N)
*c*	The number of total codewords
*a*	The number of data codewords
*r*	The capacity of correction in the QR code
*b*	The block of the QR code
→	Generation
Area Q	The module collection of QR code
Stacking(·)	The decoding operation of the VCS

**Table 2:** The correcting ability (part of the ISO [48]).

Version	Error Correction Level	Number of Codewords (c,a,r)
4	L	(100, 80, 10)
	M	(50, 32, 9)
	Q	(50, 24, 13)
	H	(25, 9, 8)
5	L	(134, 108, 13)
	M	(67, 43, 12)
	Q	(33, 15, 9)
		(34, 16, 9)
	H	(33, 11, 11)
		(34, 12, 11)

*c*, *a* and *r* are the numbers of total codewords, data codewords, and the capacity of correction, respectively.

**Table 3:** The operation of stacking.

Pixel Color-1	Pixel Color-2	Stacking
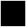	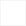	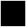
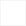	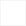	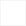
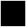	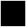	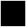

**Table 4:** The decoding test of the restored image.

The QR Code	Correct Decoding with a Standard Decoder
Figure 4h	yes
Figure 4i	no
Figure 4j	no
Figure 4k	no
Figure 4l	no

**Table 5:** The number of the incorrect codeword.

Operation	Number of Wrong Codewords (Block 1, Block 2, Block 3, Block 4)
Stacking ($S_{1},\;\cdots ,\;S_{6}$)	(0, 0, 0, 0)
Stacking ($S_{1},\;\cdots ,\;S_{4},\;S_{6}$)	(0, 13, 0, 0)
Stacking ($S_{1},\;\cdots ,\;S_{3},\;S_{6}$)	(0, 13, 0, 12)
Stacking ($S_{1},\;S_{2},\;S_{6}$)	(0, 25, 0, 12)
Stacking ($S_{1},\;S_{6}$)	(0, 5, 25, 12)

**Table 6:** The comparison result.

	Pixel Expansion	Meaningful Shares	RecoveredImage withErrors
[41]	yes	no	no
[42]	no	yes	yes
[43]	no	yes	yes
[45]	yes	yes	yes
[46]	no	yes	yes
Proposed scheme	no	no	no

## Data Availability

The data used to support the findings of this study are included in the article.
